# A novel detachable over-the-scope clip system for the management of esophageal inlet perforation secondary to endoscopic ultrasonography procedures

**DOI:** 10.1093/gastro/goaf043

**Published:** 2025-05-23

**Authors:** Benhua Wu, Lisheng Wang, Wenbiao Chen

**Affiliations:** Department of Gastroenterology, Shenzhen People’s Hospital, The Second Clinical Medical College, Jinan University; The First Affiliated Hospital, Southern University of Science and Technology, Shenzhen, P. R. China; Department of Gastroenterology, Shenzhen People’s Hospital, The Second Clinical Medical College, Jinan University; The First Affiliated Hospital, Southern University of Science and Technology, Shenzhen, P. R. China; Department of Gastroenterology, Shenzhen People’s Hospital, The Second Clinical Medical College, Jinan University; The First Affiliated Hospital, Southern University of Science and Technology, Shenzhen, P. R. China

## Introduction

Esophageal inlet perforations pose significant challenges due to the complex anatomical features of the esophageal entrance, making surgical closure difficult, and often limiting surgical options to drainage procedures only [1]. Endoscopic ultrasonography, which involves an oblique-viewing lens and a longer inflexible distal end, may cause perforation of the esophageal inlet during initial entry through the oral cavity [2]. Recent studies have indicated that endoscopic vacuum therapy can be used to manage esophageal perforations [3]. However, this technique also involves frequent sponge material changes under endoscopy, increasing costs and extending durations of treatment. The over-the-scope clip (OTSC) can be used to close gastrointestinal defects up to 2 cm in size [4, 5]. However, it does not detach on its own and cannot be retrieved endoscopically, limiting its use to less restrictive areas such as the lower esophagus, stomach, duodenum, and colon. Using the OTSC at the esophageal entrance, where space is limited, may result in stenosis, and there have been no prior reports of its use for this indication. Here, we provided a novel OTSC, the important feature of this equipment is detachable (Ningbo SensCure Biotechnology Co., Ltd, P. R. China). We performed the detachable OTSC to seal an esophageal entrance perforation caused by endoscopic ultrasound, and achieved secure closure and subsequent removal of the OTSC device after the perforation was healed, preventing stenosis. The successful use of detachable OTSC may provide a new therapeutic approach for the treatment of esophageal perforation.

## Case report

**Figure 1.**
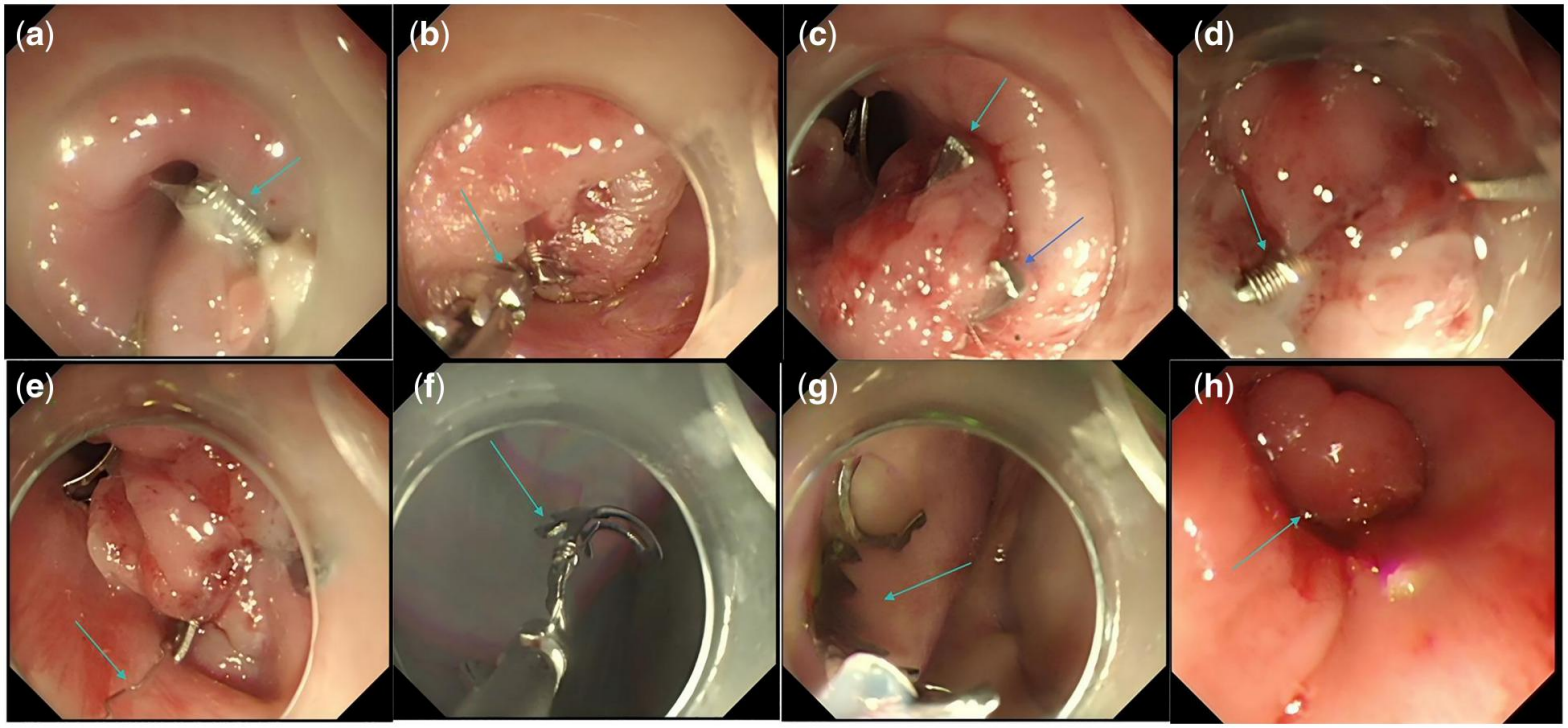
Dismantling process of detachable over-the-scope clip device in esophagus. (A) The traction ring was too small to locate, but the first binding thread (light blue arrow) was easily identified. (B) The first binding thread (light blue arrow) was grasped with biopsy forceps and pulled out through the endoscope's working channel. (C) After removal of the first binding thread, one end of the clip was successfully detached (light blue and dark blue arrows). (D) The other binding thread (light blue arrow), located opposite the first, was also found. (E) The other binding thread (light blue arrow) was pulled out using biopsy forceps. (F) After untying the second binding thread, half of the clip was successfully removed. (G) The remaining half of the clip was retrieved using a snare. (H) After removal of the clip, a mucosal bulge was still visible, but the endoscope passed smoothly without resistance.

A 49-year-old male underwent an endoscopic ultrasound for a submucosal duodenal tumor at an outpatient clinic that resulted in a perforation at the esophageal entrance, extending into the mediastinum. Following the incident, a gastroscope revealed a 1.3 cm × 1.3 cm esophageal tear located 16 cm from the incisors. We initially attempted to close this perforation using titanium clips, but the procedure failed because of space constraints in this area. Next, we successfully sealed the perforation using a novel detachable OTSC device (Supplementary Figures 1 and 2 and Supplementary Video 1). After the procedure, the patient was afebrile, and white blood cell count and inflammatory markers remained within normal ranges. After 5 days of fasting, the patient was transitioned to a liquid diet and was then discharged without any discomfort. After discharge, the patient was able to consume a liquid diet but had more difficulty with semi-solid and solid foods. The patient was readmitted 1 month after discharge for a gastroscopic review, which demonstrated that the esophageal entrance perforation had healed. However, the OTSC had not detached, and a narrowing at the esophageal entrance prevented a 9.9 mm diameter gastroscope from passing. With the assistance of a transparent cap on the tip of the gastroscope, the detachable OTSC was successfully removed by first capturing its two traction rings with biopsy forceps and then extracting it (Figure 1). Following extraction, the 9.9 mm diameter endoscope was able to pass through smoothly. On the second post-operative day, the patient was able to resume liquid and semi-solid diets without discomfort. Three days later, after tolerating solid food, the patient was discharged.

## Discussion and conclusions

Although the traditional OTSC was designed to be available for permanent implantation, there are occasions upon which the OTSC needs to be removed such as ‘adverse events after over-the-scope clip implantation’ (i.e. ulceration, stenosis, obstruction) and ‘misplacement’ [5, 6]. A meta-analysis of pooled success rates of traditional OTSC removal was 89%, and these methods for removal may lead to minor bleeding, superficial thermal damage, and superficial mucosal tears [7]. The innovative detachable OTSC, designed for easy removal after healing, is constructed with two thin metal wires that tie its separable parts together. This design permits the OTSC to be separated by pulling out of the binding thread or the traction loops, avoiding the more challenging need to dissolve its nitinol structure using argon plasma coagulation or other methods. Our case had demonstrated that the innovative detachable OTSC not only effectively seals perforations at the entrance to the esophagus but also allows for straightforward removal and extraction post-healing, thus preventing stricture. Further research is necessary to confirm the reliability and safety of the innovative detachable OTSC.

In conclusion, the detachable OTSC device thus effectively sealed an esophageal perforation and could be extracted endoscopically after healing, unlike other perforation treatment options. The OTSC may help improve outcomes for perforations at the esophageal entrance.

## Supplementary Material

goaf043_Supplementary_Data
