# Supplementary figures and images for: A novel detachable over-the-scope clip system for the management of esophageal inlet perforation secondary to endoscopic ultrasonography procedures
Source: Gastroenterol Rep (Oxf). 2025 May 23;13:goaf043. doi: 10.1093/gastro/goaf043 (PMC12102062; doi:10.1093/gastro/goaf043)

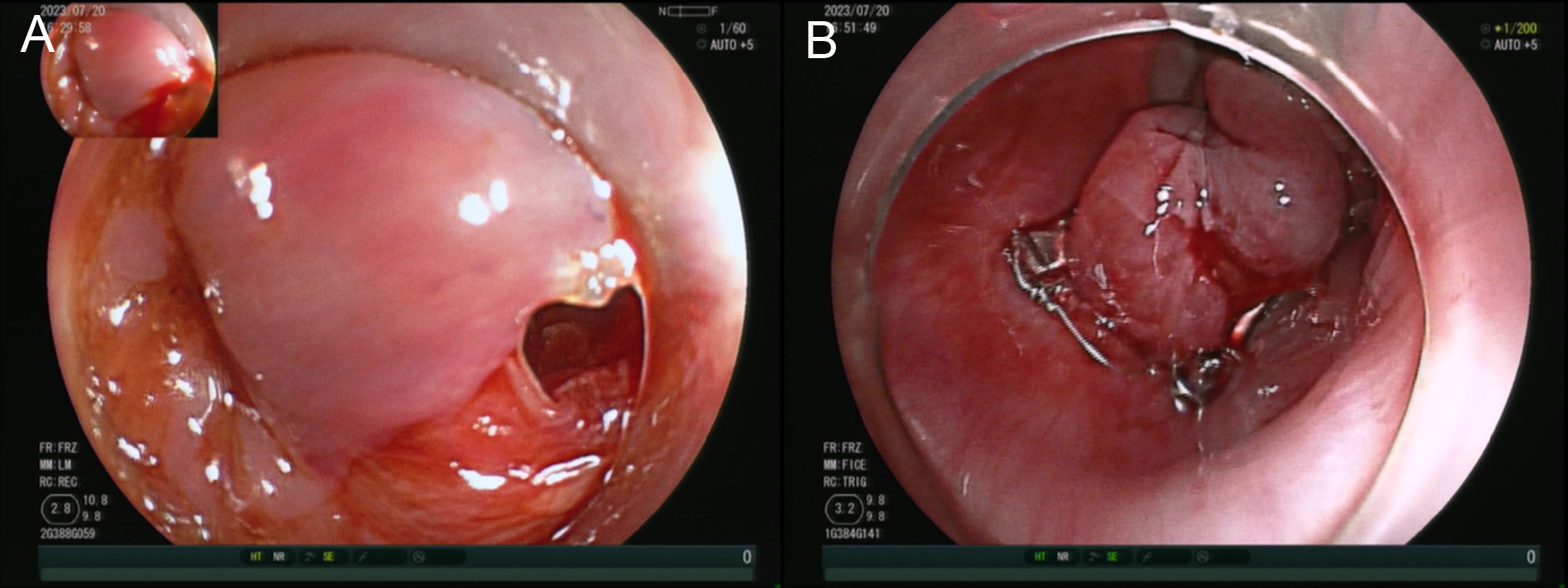

Supplement: goaf043_Supplementary_Data [file goaf043_supplementary_data.zip › Suppl Figure 1 20240422.tif]
